# A Systematic Review and a Meta-Analysis Comparing Prophylactic and Therapeutic Low Molecular Weight Heparins for Mortality Reduction in 32,688 COVID-19 Patients

**DOI:** 10.3389/fphar.2021.698008

**Published:** 2021-09-02

**Authors:** Riccardo Giossi, Danilo Menichelli, Arianna Pani, Elena Tratta, Alessandra Romandini, Rossana Roncato, Alessandro Nani, Paolo Schenardi, Erika Diani, Veronica Andrea Fittipaldo, Alessio Farcomeni, Francesco Scaglione, Daniele Pastori

**Affiliations:** ^1^ Postgraduate School of Clinical Pharmacology, Department of Oncology and Hemato-Oncology, University of Milan, Milan, Italy; ^2^ Department of Clinical Internal, Anesthesiological and Cardiovascular Sciences, Sapienza University of Rome, Rome, Italy; ^3^ Department of Oncology and Hemato-Oncology, University of Milan, Milan, Italy; ^4^ Central Pharmacy, ASST Spedali Civili, Brescia, Italy; ^5^ Experimental and Clinical Pharmacology Unit, Centro di Riferimento Oncologico di Aviano (CRO), IRCCS, Aviano, Italy; ^6^ Pharmacy Unit, ASST Papa Giovanni XXIII, Bergamo, Italy; ^7^ Oncology Department, Mario Negri Institute for Pharmacological Research IRCCS, Milano, Italy; ^8^ Department of Economics and Finance, University of Rome “Tor Vergata”, Rome, Italy; ^9^ Department of Laboratory Medicine, ASST Grande Ospedale Metropolitano Niguarda, Milan, Italy

**Keywords:** SARS-CoV2, COVID-19, mortality, heparin, LMWH, bleeding, thromboembolism

## Abstract

**Background:** Antithrombotic treatment, including low molecular weight heparin (LMWH) or unfractionated heparin (UFH), has been proposed as a potential therapy for coronavirus disease 2019 (COVID-19) to lower diffuse intravascular clotting activation. However, it is unclear whether prophylactic or therapeutic doses have similar efficacy in reducing mortality.

**Methods:** We performed a systematic review (PROSPERO registration CRD42020179955) and meta-analysis including observational cohort studies and randomized controlled trials (RCT) evaluating the effectiveness of heparins (either LMWH, UFH, or fondaparinux) in COVID-19 patients. Heparin treatment was compared to no anticoagulation. A subgroup analysis on prophylactic or therapeutic doses compared to no anticoagulation was performed. Prophylactic dose was also compared to full dose anticoagulation. Primary endpoint was all-cause mortality. Secondary endpoints were major bleeding and length of hospital stay (LOS).

**Results:** 33 studies (31 observational, 2 RCT) were included for a total overall population of 32,688 patients. Of these, 21,723 (66.5%) were on heparins. 31 studies reported data on all-cause mortality, showing that both prophylactic and full dose reduced mortality (pooled Hazard Ratio [HR] 0.63, 95% confidence interval [CI] 0.57-0.69 and HR 0.56, 95% CI 0.47-0.66, respectively). However, the full dose was associated with a higher risk of major bleeding (Odds Ratio [OR] 2.01, 95% CI 1.14–3.53) compared to prophylactic dose. Finally, LOS was evaluated in 3 studies; no difference was observed between patients with and without heparins (0.98, −3.87, 5.83 days).

**Conclusion:** Heparin at both full and prophylactic dose is effective in reducing mortality in hospitalized COVID-19 patients, compared to no treatment. However, full dose was associated with an increased risk of bleeding.

**Systematic Review Registration**: https://clinicaltrials.gov/, identifier CRD42020179955

## Introduction

Severe acute respiratory syndrome coronavirus 2 (SARS-CoV-2) causing coronavirus disease 2019 (COVID-19) was firstly detected in Wuhan, China, in December 2019 and rapidly spread worldwide with recurrent infection waves. COVID-19 primarily involves the respiratory tract leading, in the more severe cases, to interstitial pneumonia and acute respiratory distress syndrome (ARDS) requiring intensive care unit admission and ventilatory support (Krishnan et al., 2021). Risk factors associated with severe ARDS and poor prognosis are the coexistence of cardiovascular and noncardiovascular comorbidities such as diabetes, hypertension, previous cerebrovascular and cardiovascular disease, chronic obstructive pulmonary disease (COPD), and male sex (Del Sole et al., 2020; Gómez Antúnez et al., 2020; Jordan et al., 2020; Wu et al., 2020; Zhang et al., 2020).

Among pathophysiological mechanisms, it has been shown that COVID-19 may cause a diffuse pulmonary intravascular coagulopathy associated with systemic inflammation promoting an extensive alveolar and interstitial lung inflammation leading to local microthrombosis (McGonagle et al., 2020). COVID-19 may also be implicated in platelet activation and arterial dysfunction leading to arterial thrombosis such as myocardial infarction (Violi et al., 2020). According to these mechanisms, disproportionally high D-dimer levels were also described (Del Sole et al., 2020), which were associated with higher mortality (Soni et al., 2020; Zhang et al., 2020).

Following these observations, empirical treatments targeting inflammatory pathways have been proposed, such as tocilizumab and hydroxychloroquine (Cavalcanti et al., 2020; Ip et al., 2020; Salama et al., 2021), with divergent results among studies (Abubakar et al., 2020; Geleris et al., 2020; Jordan et al., 2020; Lauriola et al., 2020; Abdulrahman et al., 2021; Veiga et al., 2021). Currently, remdesivir is the only recommended drug in virtue of its ability to reduce the length of hospital stay in COVID-19 patients (Beigel et al., 2020).

Besides, therapies aimed at reducing the procoagulant phenotype of these patients, such as low molecular weight heparins (LMWH), have been investigated (Albani et al., 2020; Nadkarni et al., 2020), but the evidence is limited, and guidelines are not completely concordant (Marietta et al., 2020; Moores et al., 2020; Spyropoulos et al., 2020).

However, the real clinical benefit of LMWH in this setting is not known, and it is unclear whether patients should be kept at prophylactic or therapeutic doses of LMWH. For this reason, we conducted a systematic review and meta-analysis of the literature to evaluate the effectiveness of heparin compared to no anticoagulant treatment in reducing overall mortality. Also, we evaluated major bleeding and length of hospital stay in patients treated with heparin as secondary endpoints.

## Methods

### Searches Strategy and Study Selection

From April 30, 2020, to June 22, 2021, we monthly researched MEDLINE (Pubmed), Embase, Cumulative Index to Nursing and Allied Health Literature (CINAHL) (EBSCO host), Cochrane Central Register of Controlled Trials (CENTRAL 2020) in the Cochrane Library, and WHO Global Index Medicus for potentially relevant results. The search strategy included “enoxaparin,” “fondaparinux,” and “COVID-19” as keywords and is detailed in Supplementary Material S1. No filters were applied. The search strategy was performed according to PRISMA guidelines. Websites of regulatory agencies and pharmaceutical companies of included treatments were searched, too. We included records in English and Italian.

Initial inclusion criteria were as follows: 1) full-text articles of randomized controlled trials (RCTs) or non-RCT or observational studies; 2) the study condition was COVID-19 in adult patients (older than 18 years); 3) the intervention was enoxaparin or fondaparinux with or without other concomitant therapies for COVID-19 at prophylactic or therapeutic doses; 4) the comparator was placebo or standard of care, with or without other concomitant therapies for COVID-19. Studies administering low molecular weight heparins (LMWH), unfractionated heparin (UFH), or combined anticoagulant regimens with indicated prophylactic or therapeutic dose were included. Studies investigating oral anticoagulants were excluded. Case reports, case series, studies with no comparator, commentaries, and editorials were excluded. Letters were excluded unless reported original data fulfilling our inclusion criteria. Studies without outcomes were excluded. Reviews and meta-analyses were excluded, too.

Retrieved citations were screened by title and abstract independently by six study authors. Full texts of potentially relevant citations were assessed by two authors for final decision of inclusion or exclusion, and disagreements were solved by collegial discussion.

### Data Extraction

From the included studies, we collected data on author name, study design, mean age, sex, total patients, treatment and control arms with administered dose, the number of patients in each arm, comorbidities (hypertension, diabetes mellitus, heart failure, and malignancy), setting, D-dimer, and Sequential Organ Failure Assessment (SOFA) score, when available. All studies and outcomes data were collected in an electronic spreadsheet (Microsoft Excel).

### Study Outcomes

Included outcomes were all-cause mortality, major bleeding, and length of hospital stay (LoS).

### Study Quality and Risk of Bias Assessment

**FIGURE 1:**
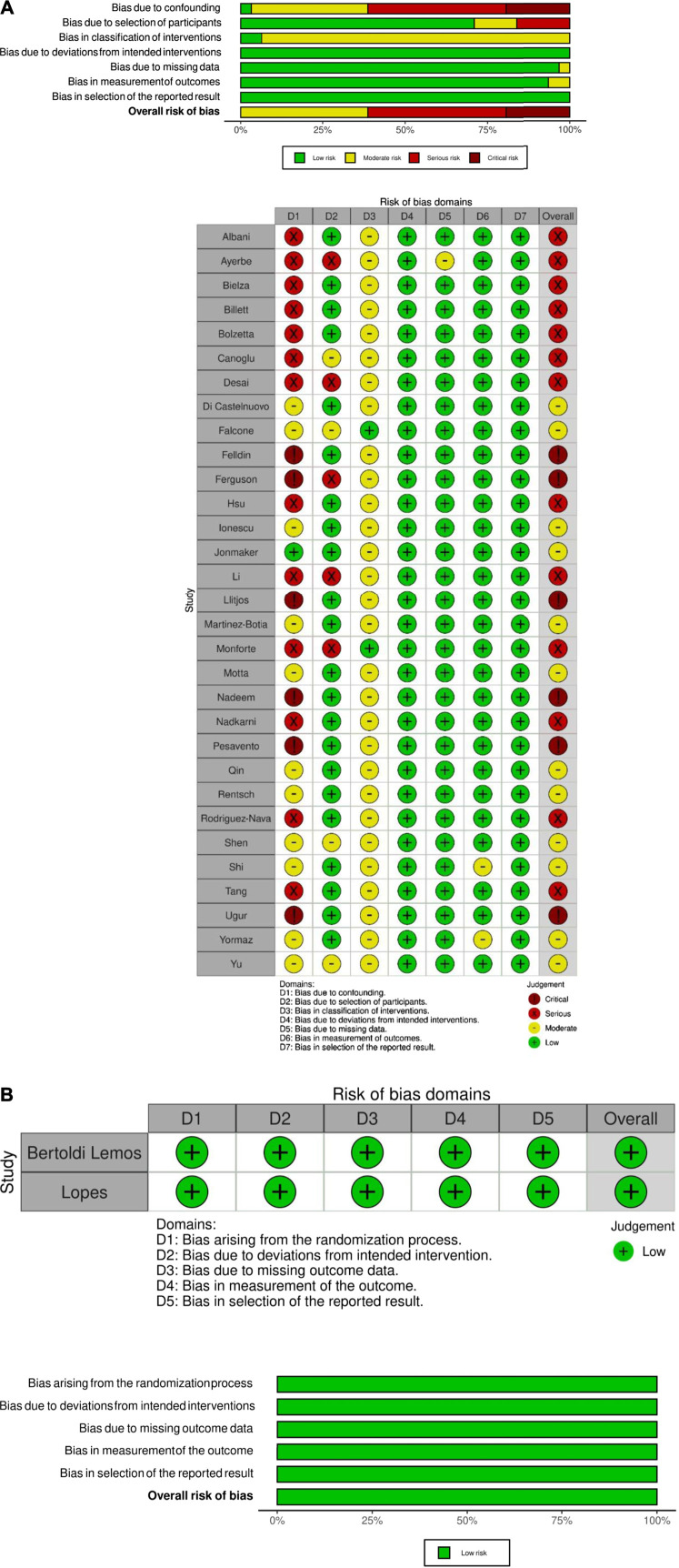
Risk of bias assessment of observational studies **(Panel A)** and randomized controlled trials **(Panel B)**.

Study quality was evaluated by two study authors (R.G. and D.M.) with Newcastle-Ottawa scales (See Supplementary Table S1). For the evaluation of adequate follow-up in Newcastle-Ottawa scales, we used a threshold of at least 28 days to avoid potential loss of observation of the outcome. Studies with a score ≥7 were considered of good quality. ROBINS-I tool was also used to evaluate the risk of bias of observational studies **(** Figure 1A; Sterne et al., 2016 **)**. RoB2 tool was used to assess risk of bias for RCTs (Figure 1B) (Sterne et al., 2019 **).** Publication bias was evaluated by funnel plots (Supplementary Figure S1).

### Statistical Analyses

A primary analysis was performed on all included studies on the basis of prophylactic, full dose, and overall treatment with enoxaparin, fondaparinux, an unspecified LMWH, UFH, or combined anticoagulant regimens compared to placebo or standard of care. Also, a comparison between prophylactic and full dose treatment was performed. For studies not reporting HR estimates, when sufficient other information was available, effects and their standard errors were approximated as described in Tierney et al. (2007). Odds ratios (OR) and their standard errors were directly calculated on the basis of the number of subjects and events per group. Meta-analyses for each endpoint separately were performed based on random effect models, using the logarithm of hazard ratios (HR) or OR as outcome. According to Higgins et al. (2009), we performed Bayesian meta-analysis with informative priors, since some analyses were based on a limited number of studies. For the LoS endpoint, we performed a meta-analysis of differences in medians, rather than in means, according to McGrath et al. (2020). Evaluation of the difference in medians is here more appropriate for two reasons: first, many studies only report information on quantiles (e.g., median and quartiles), and mapping to mean and standard deviation might be biased; secondly, LoS is skewed, and, therefore, the median is a more appropriate summary. Analyses were performed using the R software (R Development Core Team, 2018) version 3.5.1.

### Summary of Findings

Summary of findings with grading of the quality of the evidence was performed using GRADEproGDT according to the GRADE Handbook by two study authors (Zhang et al., 2020). Discrepancies were resolved by discussion.

### Study Registration and Approval

This study was registered in PROSPERO (CRD42020179955). Due to the secondary nature of the study on already published data, institutional review board (IRB) approval and patient consent were not necessary.

## Results

### Study Characteristics and Results of Individual Studies

**FIGURE 2:**
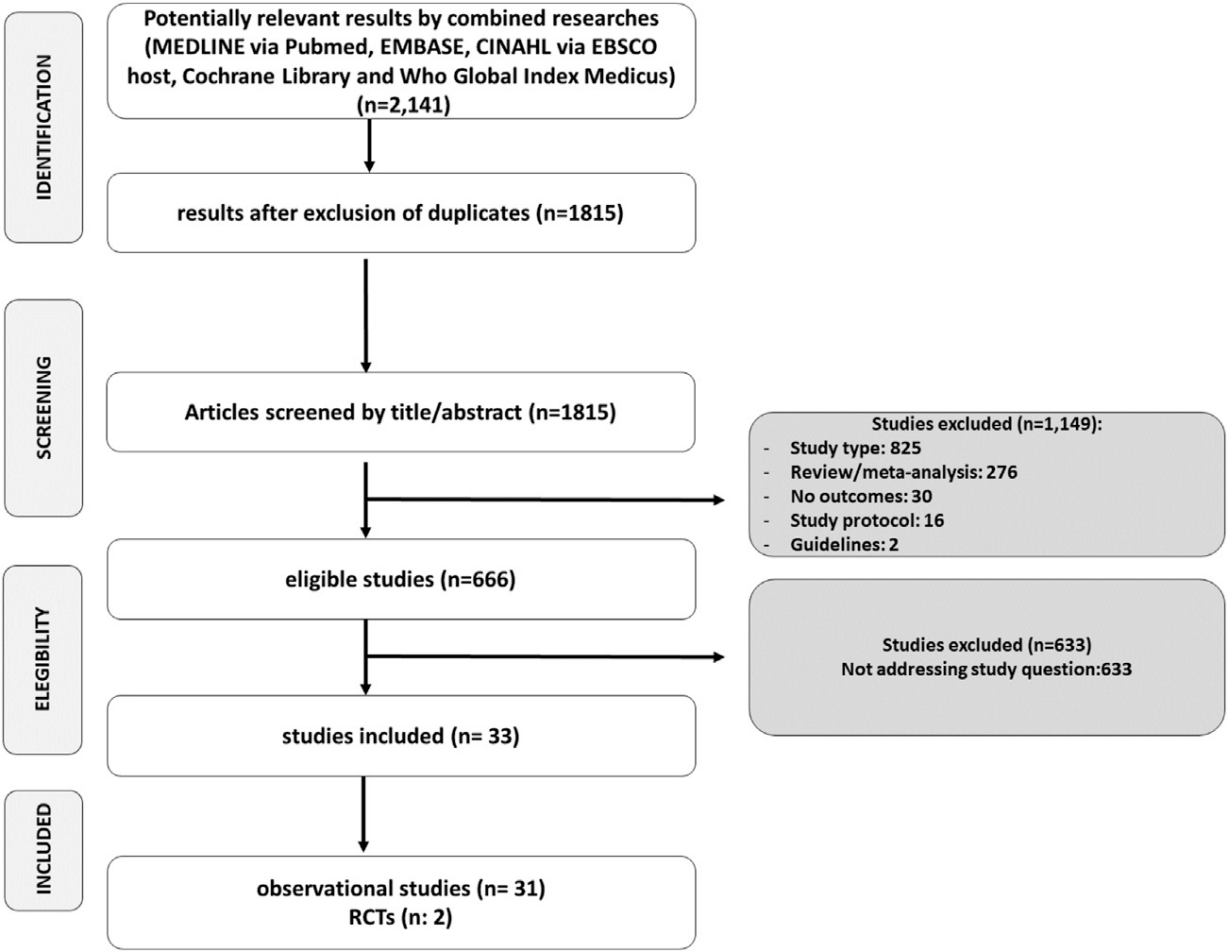
PRISMA flow diagram.

After screening, **666** potentially eligible studies were identified and were considered for detailed analysis (Figure 2); 33 studies were finally included in the meta-analysis: 31 observational studies and 2 RCTs. Included studies’ characteristics are detailed in Table 1.

**TABLE 1:** Clinical characteristics of studies included in the meta-analysis.

Author (year)	Study design	Age	Male (%)	Total patients	Heparin dose				No AC	Hypertension (%)	Diabetes (%)	Heart failure (%)	Severe Covid (%)
					Not specified	Prophylactic	Intermediate	Full					
Albani et al. (2020)	R—cohort	70.4	65.8	1,403	799	—	—	—	604	35.1	19.0	—	—
Ayerbe et al. (2020)	R—cohort	67.6	60.5	2,019	1,734	—	—	—	285	—	—	—	—
Lemos et al. (2020)	RCT	56.5	80.0	20.0	—	10	—	10	—	35.0	35.0	—	100.0^a^
Bielza et al. (2021)	R—cohort	87.0	35.4	502	502	—	—	-	128	60.4	15.2	10	74.2^b^
Billett et al. (2020)	R—cohort	—	52.6	3,625	—	1,544	—	163	639	-	-	—	8.6^a^
Bolzetta et al. (2021)	R—cohort	84.1	38.1	81	—	57	—	24	—	63.0	24.7	3.7	—
Canoglu and Saylan (2020)	R—case control	—	62.3	154	—	98	—	56	—	—	—	—	—
Desai et al. (2020)	R—cohort	64.8	66.1	575	240	—	—	—	335	43.1	20.0		—
Di Castelnuovo et al. (2021)	R—cohort	67.1	61.6	2,574	1,804	—	—	—	770	71.7	28.2	—	—
Falcone et al. (2020)	P—cohort	70.0	76.2	315	244	—	—	—	71	46.0	16.5	—	17.5^a^
Felldin et al. (2020)	R—cohort	56.0	57.0	53	27	—	—	—	26	54.7	30.2	3.8	32.0^c^
Ferguson et al. (2020)	R—cohort	63.6	55.3	141	—	95	—	46	—	—	24.1	—	—
Hsu et al. (2020)	R—cohort	60.2	55.3	452	—	377	—	48	27	—	36.9	—	—
Ionescu et al. (2020)	R—cohort	64.5	48.5	3,480	—	2,121	—	998	361	52.1	29.0	7.8	18.5^a^
Jonmarker et al. (2020)	R—cohort	61.0	82.2	152	—	67	48	37	—	45.4	16.5	—	—
Li et al. (2020)	R—case control	63.8	64.0	71	—	28	—	28	—	68.0	64.0	—	39.0^a^
Llitjos et al. (2020)	R—cohort	68.0	77.0	26.0	-	8	—	18	—	85.0	—	—	100.0^a^
Lopes et al. (2021)	RCT	56.6	60.0	615	—	311	—	304	—	49.5	24.5	2.5	6.5
Martínez-Botía et al. (2021)	R—cohort	—	60.0	2,035	342	—	—	—	342	—	—	—	—
d'Arminio Monforte et al. (2020)	P—cohort	60.0	63.0	539	—	355	—	—	184	46.4	17.6	—	48.6^d^
Motta et al. (2020)	R—cohort	64.7	58,8	374	—	299	—	75	—	—	31.6	—	11.8
Nadeem et al. (2021)	R—case control	50.7	86.6	74	—	34	—	40	—	28.1	47.0	—	81.8
Nadkarni et al. (2020)	R—cohort	65.0	56.0	4,389	—	1,959	—	900	1,530	34.8	22.6	8.3	10.6^a^
Pesavento et al. (2020)	R—cohort	71.0	55.9	324	—	240	—	84	—	—	—	—	—
Qin et al. (2021)	P—cohort	60.0	48.0	749	—	109	—	77	—	—	—	—	—
Rentsch et al. (2021)	P—cohort PW	68.2	93.4	4,297	—	3,627	—	-	670	67.8	42.8	10.5	15.2^e^
Rodriguez-Nava et al. (2021)	R—case control	68.0	58.1	313	—	175	—	91	21	70.9	44.7	—	78.9^c^
Shen et al. (2021)	R—cohort	64.0	49.3	525	—	120	—	—	405	37.3	17.7	10.5	15.1^f^
Shi et al., (2020)	R—cohort	69.0	64.3	42	—	21	—	—	21	30.1	19.0	—	100.0^e^
Tang et al. (2020)	R—case control	65.1	59.7	449	—	99	—	—	350	39.4	20.7	—	100.0^f^
Ugur et al. (2021)	R—cohort	50.6	54.2	1,251	253	—	—	—	998	—	—	—	—
Yormaz et al. (2020)	R—cohort	54.4	68.8	96	—	48	—	—	48	36.5	25.0	—	—
Yu et al. (2021)	R—cohort	61.8	57.3	973	—	764	—	165	—	44.8	46.5	6.5	29.6

ICU, intensive care unit; N/A, not applicable; P, prospective; PW, propensity weighted; R, retrospective.

a Defined as mechanical ventilation/intubation.

b Defined according to the World Health Organization 2020 Clinical management of severe acute respiratory infection (SARI) when COVID-19 disease is suspected, as temperature >38°, systolic blood pressure <100 mm Hg, heart rate >100 beats per minute, basal saturation less than 90%, respiratory rate >30 per minute, or altered level of consciousness.

c Defined according to COVID-19 Treatment Guidelines Panel of the National Institutes of Health (SpO2 <94% on room air at sea level, PaO_2_/FiO_2_ <300 mmHg, RR > 30 bpm, or lung infiltrates >50%).

d Defined as respiratory rate (RR) < 24/min, SO_2_ < 92% or PaO_2_/FiO_2_ <300 mmHg.

e Defined as any of the following: shortness of breath, RR ≥ 30 bpm; SO_2_ ≤ 93% (at rest); PaO_2_/FiO_2_≤300 mmHg; pulmonary inflammation that progresses significantly within 24–48 h >50%.

f Severe COVID-19 was defined according to the Diagnosis and Treatment Plan of COVID-19 suggested by National Health Commission of China (RR ≥ 30 breaths/min; SO_2_ ≤ 93% at rest; PaO_2_/FiO_2_ ≤ 300 mm Hg.

Quality assessment showed a general low quality of observational studies, with only five studies included having a score ≥7 (Figure 1; Supplementary Table S1).

A total of 32,688 patients with COVID-19 were included, of whom 21,723 (66.5%) were on treatment with heparins (including LMWH and UFH) or fondaparinux, and 10,965 (33.5%) were not treated with anticoagulants. Men were the most represented with the prevalence ranging from 35.4% to 80.0% among studies, while the range of age among studies was 55.0–87.0 years. The rate of patients with hypertension and diabetes ranges among 28.1–85.0% and 15.2–64.0%, respectively. The proportion of patients with severe COVID-19 ranged between 6.5 and 100%.

The use of heparins was highly heterogeneous, and a detailed definition of treatments for each included study is reported in Supplementary Table S2.

### All-Cause Mortality

Overall, 31 studies reported data on all-cause mortality including 32,550 patients and 4,789 deaths (Table 2).

**TABLE 2:** Number of events according to each endpoint in patients treated or not with heparin.

Endpoints	Number of studies	Total number of patients	Total number of events
All-cause mortality^a^	31	32,550	4,789
Major bleeding^b^	4	12,691	426

a Six studies reported only hazard ratio.

b One study reported only odds ratio.

**FIGURE 3:**
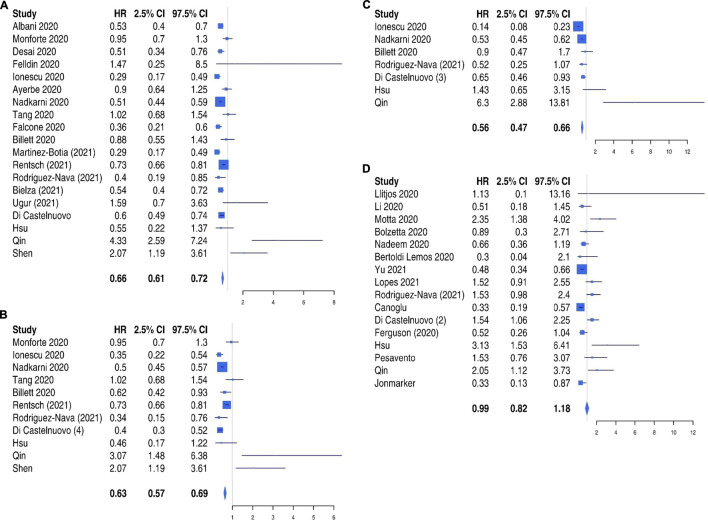
All-cause mortality risk in patients treated with heparin: overall analysis **(Panel A)**, prophylactic dose **(Panel B),** and full dose **(Panel C)** compared to no treatment and comparison between full dose and prophylactic dose **(Panel D)**.

In the overall analysis (Figure 3A), heparin treatment reduced the risk of all-cause mortality (pooled HR 0.66, 95% CI 0.61–0.72). These results were confirmed by sensitivity analysis performed on both prophylactic (Figure 3B) and full dose of heparin (Figure 3C) (pooled HR 0.63, 95% CI 0.57–0.69 and pooled HR 0.56, 95% CI 0.47–0.66, respectively).

Furthermore, a direct comparison of full and prophylactic dose (Figure 3D) was performed, and no difference was found between the two strategies in the reduction of mortality (HR 0.99, 95% CI 0.82–1.18).

### Major Bleeding

Four studies reported data on major bleeding including 12,691 patients and 426 MBs (Table 2). In the overall analysis (Supplementary Figure S2A), no difference was observed regarding the occurrence of major bleeding in patients treated or not with heparin (Odds Ratio [OR] 0.88, 95% CI 0.77–1.08). Both prophylactic (Supplementary Figure S2B) and full dose (Supplementary Figure S2C) of heparin did not significantly increase bleeding risk when compared to no treatment (OR 0.81, 95% CI 0.66–1.0 and OR 1.55, 95% CI 0.98–2.44, respectively). A full dose was associated with an increased risk of bleeding compared to prophylactic (OR 2.01, 95% CI 1.14–3.53) (Supplementary Figure S2D).

### Length of Hospital Stay

Six studies reported data on length of hospital stay including 2,233 patients, of whom 1,056 were not treated with heparin. The difference of length of stay for all heparin treatment compared to no treatment (Supplementary Figure S3A) was 0.98 (−3.87, 5.83), while prophylactic dose reduced the length of stay compared to no treatment (Supplementary Figure S3B) (−2.38, −3.14, −1.61). A full dose was associated with higher length of stay compared to prophylactic dose (2.83, 0.42, and 5.25).

A summary of pooled results of meta-analysis according to each endpoint are shown in Figure 2, and a summary of findings according to GRADE guidelines is reported in Table 3.

**TABLE 3:** Summary of findings and grading quality of the evidence.

Outcomes	Estimate of the effect (95% CI)	No of participants (studies)	Certainty of the evidence (GRADE)	Comments
Heparin overall treatment compared to no treatment				
All-cause death	HR 0.66 (0.61–0.72)	27,889 (19 observational studies)	⊕⊕◯◯ LOW^a^ ^,^ ^b^	The evidence suggests overall Heparin treatment reduces all-cause mortality
Major Bleeding	OR 0.88 (0.72–1.08)	12,691 (4 observational studies)	⊕⊕◯◯ LOW^a^ ^,^ ^c^	The evidence suggests that overall Heparin treatment results in little to no difference in major Bleeding
Length of Stay	MD 0.98 Days higher (3.87 lower to 5.83 higher)	1,541 (3 observational studies)	⊕◯◯◯ VERY LOW^a^ ^,^ ^b^ ^,^ ^d^	Overall Heparin treatment may have little to no effect on Length of Stay but the evidence is very uncertain
Heparin prophylactic treatment compared to no treatment				
All-cause death	HR 0.63 (0.57–0.69)	16,989 (11 observational studies)	⊕⊕◯◯ LOW^a^ ^,^ ^b^	Prophylactic Heparin treatment may reduce all-cause mortality
Major Bleeding	OR 0.81 (0.66–1.00)	10,793 (4 observational studies)	⊕⊕◯◯ LOW^a^ ^,^ ^e^	Prophylactic Heparin treatment may result in little to no difference in Major Bleeding
Length of Stay	MD 2.40 Days lower (3.14 lower to 1.61 lower)	138 (2 observational studies)	⊕◯◯◯ VERY LOW^a^ ^,^ ^b^ ^,^ ^d^	Prophylactic Heparin treatment may reduce Length of Stay but the evidence is very uncertain
Heparin full dose treatment compared to no treatment				
All-cause death	HR 0.56 (0.47–0.66)	6606 (7 observational studies)	⊕⊕◯◯ LOW^a^ ^,^ ^b^	Heparin full dose treatment may reduce all-cause death
Major Bleeding	OR 1.55 (0.98–2.44)	3,789 (2 observational studies)	⊕⊕◯◯ LOW^a^ ^,^ ^f^	The evidence is uncertain and suggests that heparin full dose treatment may not increase major bleeding
Heparin full dose compared to prophylactic treatment				
All-cause death	HR 0.99 (0.82–1.18)	4,524 (14 observational studies; 2 RCT)	⊕⊕◯◯ LOW^a^ ^,^ ^b^	The evidence suggests that full dose heparin treatment results in little to no difference in all-cause mortality compared to prophylactic dose
Major Bleeding	OR 2.01 (1.14–3.53)	1,183 (3 observational studies; 1 RCT)	⊕⊕◯◯ LOW^a^ ^,^ ^d^	The evidence suggests that full dose heparin treatment may result in an increase in major bleeding compared to prophylactic dose
Length of Stay	MD 2.83 days higher (0.42 higher to 5.25 higher)	692 (2 observational studies; 1 RCT)	⊕◯◯◯ VERY LOW^a^ ^,^ ^b^ ^,^ ^d^	The evidence is very uncertain about the effect of full dose heparin treatment on length of Stay

a Overall serious risk of bias across studies, mainly due to confounding.

b Unexplained inconsistency.

c Imprecision due to CI that include potential benefit and harm.

d Imprecision due to limited sample size.

e Imprecision due to CI that include non-significance and potential benefit.

f Imprecision due to large CI that include non-significance and substantial harm.

Summary of findings are presented for heparin overall treatment, prophylactic treatment, and full dose treatment. CI = confidence interval; HR = hazard ration; OR = odds ratio; MD = mean difference; RCT = randomized controlled trial.

## Discussion

The main finding of our meta-analysis is that heparin treatment (either enoxaparin, other LMWH, or UFH) significantly reduced in-hospital mortality in COVID-19 patients. We found that both prophylactic and therapeutic doses of heparin were similarly associated with a reduced mortality, with the advantage of a lower bleeding risk in the group of patients treated with prophylactic dose.

Given the similar effectiveness profile, our data does not support an extensive use of full-dose anticoagulation in all hospitalized COVID-19 patients and suggests that prophylactic dose should represent the first-choice treatment, especially in patients with high bleeding risk.

Our study showed no difference in overall mortality between prophylactic and full dose of anticoagulation. This evidence is consistent with National Institutes of Health (NIH) guidelines (Hindricks et al., 2021), which suggest that a prophylactic dose of anticoagulants should be administered to hospitalized patients with SARS-CoV-2 infection unless contraindicated (level of evidence AIII), preferring LMWH over oral anticoagulants. In addition, NIH guidelines do not support the use of therapeutic dose of antithrombotic treatment due to lack of evidence (Hindricks et al., 2021). Our study confirms the beneficial effect of prophylactic LMWH in reducing all-cause mortality which should be preferred to therapeutic LMWH filling up current NIH guidelines. Also, CHEST guidelines recommend the use of a prophylactic dose of heparins over intermediate and therapeutic dose of heparins in severely ill patients with COVID-19 (Moores et al., 2020). Of note, CHEST guidelines support the use of LMWH or fondaparinux over UFH, and the use of heparins over direct oral anticoagulants (Moores et al., 2020).

Recently, the World Health Organization (WHO) guidelines suggested the use of prophylactic over therapeutic LMWH given the very low evidence on therapeutic LMWH in lowering mortality and pulmonary embolism, along with an increased risk of major bleedings (Jakobsen et al., 2019).

The studies included in our analysis had a variable proportion of severe COVID-19, suggesting that LMWH administration may be useful in patients with both mild/moderate and severe COVID-19. This finding is consistent with the International Society of Thrombosis and Haemostasis (ISTH) interim guidance, which suggests that the use of LMWH should be used in all hospitalized COVID-19 patients (Thachil et al., 2020).

However, it is reassuring that heparin treatment was not globally associated with an increased risk of major bleeding, suggesting that it can be safely administered in this clinical setting. Nevertheless, this analysis was performed on a limited number of patients and cannot be considered as definitive. According to our findings, the American Society of Hematology (ASH) recently suggested the use prophylactic-intensity over intermediate and therapeutic-intensity anticoagulation in patients with COVID-19-related acute illness without established or suspected VTE, but this recommendation was based on very low certainty of evidence (Cuker et al., 2021).

Three clinical trials, the Randomized, Embedded, Multi-factorial Adaptive Platform Trial for Community-Acquired Pneumonia (REMAP-CAP, NCT02735707) Therapeutic Anticoagulation, Accelerating COVID-19 Therapeutic Interventions and Vaccines-4 (ACTIV-4, NCT04505774) Antithrombotics Inpatient, and Antithrombotic Therapy to Ameliorate Complications of COVID-19 (ATTACC, NCT04372589), are ongoing, and partial results were available in a press release, which reported an interim analysis on 1000 COVID-19 patients (Cuker et al., 2021; National Institutes of Health, 2021), showing that full-dose anticoagulation in patients with moderate COVID-19, as defined by patients who did not need mechanical ventilation or intensive care unit admission, seems to reduce the need for mechanical ventilation or other supportive interventions, with similar safety than prophylactic dose (National Institutes of Health, 2021).

As a secondary endpoint, we analyzed the association between heparin use and length of stay. We found that a prophylactic dose of heparin seemed to reduce length of stay: this may be caused by the clinical choice of higher over prophylactic doses in patients with severe illness or admitted in intensive care unit. Indeed, in the included studies, this choice was often based on disease severity, with severe patients receiving more frequently the full dose anticoagulation.

There are still some open issues not assessed in this study, such as the case whether the administration of oral anticoagulants may be as effective as the subcutaneous/intravenous one in these patients. Furthermore, we do not know if a D-dimer-based strategy guiding the dose of heparin may be more effective than a standard weight-adjusted prophylactic dose. There are also some patients experiencing acute renal failure or liver enzymes elevation during the in-hospital staying (Lei et al., 2020); thus, the effectiveness and safety of heparin in these high-risk patients should be confirmed.

### Study Limitations

Our study has some limitations. First, fondaparinux was not well represented among studies, so that these results cannot be applied to this drug with certainty. In addition, in two studies (Ionescu et al., 2020; Nadkarni et al., 2020), a small number of patients were treated with apixaban, an oral inhibitor of Xa factor, which has the same target of heparin, possibly representing a potential confounding factor.

Moreover, the quality of studies was generally unsatisfactory, with only 11 studies included having a score ≥7 in the Newcastle-Ottawa scale evaluation. The risk of bias, assessed with ROBINS-I tool, showed an overall serious risk of bias, especially due to confounding. Furthermore, very different regimens of heparin treatments were adopted.

Another aspect relates to the effectiveness of heparin according to COVID-19 severity; indeed, the proportion of severe patients was highly variable among studies, and this may affect the overall results. Further study in severe patients is needed.

Furthermore, the rationale for treatment assignment was not univocal across studies and included disease severity, D-dimer levels, and physician choice. D-dimer levels were expressed only in a limited number of studies and with high variability in the unit of measurement used. For this reason, we could not use D-dimer levels for further analyses.

## Conclusion

In conclusion, our results indicate that heparin is effective in reducing all-cause mortality in **hospitalized** COVID-19 patients compared to no treatment. We did not find a clear advantage of using therapeutic over prophylactic dose of heparin, along with an increased bleeding risk in patients treated with full dose heparin.

## Data Availability

The raw data supporting the conclusion of this article will be made available by the authors, without undue reservation.
