# Supplementary material for: A Systematic Review and a Meta-Analysis Comparing Prophylactic and Therapeutic Low Molecular Weight Heparins for Mortality Reduction in 32,688 COVID-19 Patients
Source: Front Pharmacol. 2021 Sep 2;12:698008. doi: 10.3389/fphar.2021.698008 (PMC8443784; doi:10.3389/fphar.2021.698008)
Supplement: Supplementary file 1 [file DataSheet1.docx]

**A systematic review and a meta-analysis comparing prophylactic and therapeutic low molecular weight heparins for mortality reduction in 32,688 COVID-19 patients.**

*Giossi and Menichelli et al.*

**Supplementary data**

**Supplement S1.** Search strategy

**Pubmed**

 ((((("Enoxaparin"[Mesh]) OR "Fondaparinux"[Mesh])) OR ((enoxaparin [Text Word] OR Enoxaparine[Text Word] OR Lovenox[Text Word] OR Clexane[Text Word] OR PK-10,169[Text Word] OR PK 10,169[Text Word] OR PK10,169[Text Word] OR PK-10169[Text Word] OR PK 10169[Text Word] OR PK10169[Text Word] OR EMT-967[Text Word] OR EMT 967[Text Word] OR EMT967[Text Word] OR EMT-966[Text Word] OR EMT 966[Text Word] OR EMT966[Text Word] OR fondaparinux[Text Word] OR ‘Fondaparinux Sodium’[Text Word] OR Quixidar[Text Word] OR Arixtra[Text Word])))) AND ((((("Coronavirus Infections"[Mesh]) OR "COVID-19" [Supplementary Concept]) OR "severe acute respiratory syndrome coronavirus 2" [Supplementary Concept])) OR ('Coronavirus Infection'[Text Word] OR 'Middle East Respiratory Syndrome'[Text Word] OR mers (middle east respiratory syndrome) '[Text Word] OR '2019 novel coronavirus disease'[Text Word] OR COVID19[Text Word] OR 'COVID-19 pandemic'[Text Word] OR 'SARS-CoV-2 infection'[Text Word] OR 'COVID-19 virus disease'[Text Word] OR '2019 novel coronavirus infection'[Text Word] OR '2019-nCoV infection'[Text Word] OR 'coronavirus disease 2019'[Text Word] OR 'coronavirus disease-19'[Text Word] OR '2019-nCoV disease'[Text Word] OR 'COVID-19 virus infection'[Text Word] OR '2019-nCoV'[Text Word] OR 'Wuhan coronavirus'[Text Word] OR 'SARS-CoV-2'[Text Word] OR '2019 novel coronavirus'[Text Word] OR 'COVID-19 virus'[Text Word] OR 'coronavirus disease 2019 virus'[Text Word] OR 'COVID19 virus'[Text Word] OR 'Wuhan seafood market pneumonia virus'[Text Word]))

**Embase**

#1 'enoxaparin'/exp/mj OR 'fondaparinux'/exp/mj

#2 enoxaparin OR enoxaparine OR lovenox OR clexane OR fondaparinux OR 'fondaparinux sodium' OR quixidar OR arixtra:ti,ab,kw

#3 #1 OR #2

#4 'covid 19'/exp

#5 'coronavirus infection'/exp

#6 'coronaviridae'/exp

#7 #4 OR #5 OR #6

#8 'coronavirus infection' OR 'middle east respiratory syndrome' OR 'mers (middle east respiratory syndrome)' OR '2019 novel coronavirus disease' OR covid19 OR 'covid-19 pandemic' OR 'sars-cov-2 infection' OR 'covid-19 virus disease' OR '2019 novel coronavirus infection' OR '2019-ncov infection' OR 'coronavirus disease 2019' OR 'coronavirus disease-19' OR '2019-ncov disease' OR 'covid-19 virus infection' OR '2019-ncov' OR 'wuhan coronavirus' OR 'sars-cov-2' OR '2019 novel coronavirus' OR 'covid-19 virus' OR 'coronavirus disease 2019 virus' OR 'covid19 virus' OR 'wuhan seafood market pneumonia virus' OR coronavir* OR ncov OR covid:ti,ab

#9 #7 OR #8

#10 #3 AND #9

**Cochrane Library**

#1 MeSH descriptor: [Enoxaparin] explode all trees 685

#2 MeSH descriptor: [Fondaparinux] explode all trees 119

#3 (enoxaparin OR Enoxaparine OR Lovenox OR Clexane OR fondaparinux OR ‘Fondaparinux Sodium’ OR Quixidar OR Arixtra):ti,ab,kw 2505

#4 #1 OR #2 OR #3 2505

#5 MeSH descriptor: [Coronavirus Infections] explode all trees 38

#6 (‘COVID19 virus’ OR ‘Wuhan seafood market pneumonia virus’ OR Coronavir* or nCov or covid):ti,ab,kw 166

#7 (‘COVID-19 virus disease’):ti,ab,kw 13

#8 (‘Coronavirus Infection’ OR ‘Middle East Respiratory Syndrome’):ti,ab,kw 89

#9 (‘Wuhan coronavirus’):ti,ab,kw 19

#10 (‘coronavirus disease 2019 virus’ OR ‘COVID19 virus’ OR ‘Wuhan seafood market pneumonia virus’ OR Coronavir* or nCov or covid):ti,ab,kw 166

#11 ^1-#10-#10^ 177

#12 #4 AND #11 0

**CINAHL (Ebsco)**

S1 MH Coronavirus OR MH 'Coronavirus infections'

S2 AB ‘Coronavirus Infection’ OR ‘Middle East Respiratory Syndrome’ OR ‘MERS (Middle East Respiratory Syndrome)’ OR ‘2019 novel coronavirus disease’ OR COVID19 OR ‘COVID-19 pandemic’ OR ‘SARS-CoV-2 infection’ OR ‘COVID-19 virus disease’ OR ‘2019 novel coronavirus infection’ OR ‘2019-nCoV infection’ OR ‘coronavirus disease 2019’ OR ‘coronavirus disease-19’ OR ‘2019-nCoV disease’ OR ‘COVID-19 virus infection’ OR ‘2019-nCoV’ OR ‘Wuhan coronavirus’ OR ‘SARS-CoV-2’ OR ‘2019 novel coronavirus’ OR ‘COVID-19 virus’ OR ‘coronavirus disease 2019 virus’ OR ‘COVID19 virus’ OR ‘Wuhan seafood market pneumonia virus’ OR Coronavir* or nCov or covid

S3 S1 OR S2

S4 MH enoxaparin OR MH fondaparinux OR AB (enoxaparin OR Enoxaparine OR Lovenox OR Clexane OR fondaparinux OR ‘Fondaparinux Sodium’ OR Quixidar OR Arixtra )

S5 S3 AND S4

**WHO Global Index Medicus (search.bvsalud.org/gim/)**

(‘Coronavirus Infection’ OR ‘Middle East Respiratory Syndrome’ OR ‘2019 novel coronavirus disease’ OR COVID19 OR ‘COVID-19 pandemic’ OR ‘SARS-CoV-2 infection’ OR ‘COVID-19 virus disease’):tw AND (mj :(enoxaparin or fondaparinux)

**Supplementary Table 1.** The Newcastle–Ottawa scale score for each study.

| *N°* | *Study* | *Selection* | *Comparability* | *Outcome* | *Total* |
| --- | --- | --- | --- | --- | --- |
| 1 | Albani*^2^* | 4 | 0 | 3 | 7 |
| 2 | Monforte*^3^* | 4 | 0 | 2 | 6 |
| 3 | Desai*^4^* | 4 | 0 | 2 | 6 |
| 4 | Felldin*^1^* | 3 | 0 | 1 | 4 |
| 5 | Ionescu*^5^* | 4 | 2 | 1 | 7 |
| 6 | Nadkarni*^6^* | 4 | 2 | 2 | 8 |
| 7 | Ayerbe*^6^* | 4 | 1 | 2 | 7 |
| 8 | Tang^7^ | 4 | 1 | 2 | 7 |
| 9 | Falcone^8^ | 4 | 0 | 2 | 6 |
| 10 | Billett^9^ | 4 | 0 | 2 | 6 |
| 11 | Shi^10^ | 4 | 0 | 1 | 5 |
| 12 | Yormaz^11^ | 4 | 0 | 1 | 5 |
| 13 | Yu^12^ | 4 | 0 | 1 | 5 |
| 14 | Pesavento^13^ | 4 | 0 | 0 | 4 |
| 15 | Martinez-Botia^14^ | 4 | 2 | 2 | 8 |
| 16 | Rentsch^15^ | 4 | 2 | 2 | 8 |
| 17 | Rodriguez-Nava^16^ | 4 | 0 | 1 | 5 |
| 18 | Bielza^17^ | 4 | 0 | 2 | 6 |
| 19 | Ugur^18^ | 4 | 0 | 1 | 5 |
| 20 | Di Castelnuovo^19^ | 4 | 2 | 1 | 7 |
| 21 | Hsu^20^ | 4 | 0 | 2 | 0 |
| 22 | Qin^21^ | 4 | 0 | 2 | 6 |
| 23 | Shen^22^ | 4 | 1 | 1 | 6 |
| 24 | Llitjos^23^ | 4 | 0 | 1 | 5 |
| 25 | Li^24^ | 4 | 0 | 1 | 5 |
| 26 | Motta^25^ | 4 | 0 | 1 | 5 |
| 27 | Bolzetta^26^ | 4 | 2 | 2 | 8 |
| 28 | Nadeem^27^ | 4 | 0 | 1 | 5 |
| 29 | Canoglu^28^ | 4 | 0 | 1 | 5 |
| 30 | Ferguson^29^ | 4 | 1 | 2 | 7 |
| 31 | Jonmarker^30^ | 4 | 1 | 2 | 7 |

**Supplementary Table 2. Type of endpoints and treatment arms reported in each study included in the metanalysis**

| N° | Author  (year) | All-cause  mortality | Major  Bleeding | Length of hospital stay | Definition of treatment |
| --- | --- | --- | --- | --- | --- |
| *1* | *Albani (2020)^2^* | X | - | X | *Treatment: enoxaparin cohort*  *Control arm: no treatment* |
| *2* | *Ayerbe^6^ (2020)* | X | - | - | *Treatment: heparin without information about dose or type*  *Control arm: no treatment* |
| *3* | *Bertoldi Lemos (2020)* | X | X | X | *Therapeutic anticoagulation: >4 days*  *- in patients <75 years: enoxaparin 1 mg/kg twice daily in CrCl>50 mL/min*  *enoxaparin 0.75 mg/kg twice daily in CrCl 30-50 mL/min*  *enoxaparin 1 mg/kg once daily in CrCl 10-30 mL/min*  *- in patients >75 years: enoxaparin 0.75 mg/kg twice daily in CrCl>50 mL/min*  *enoxaparin 1 mg/kg once daily in CrCl 30-50 mL/min*  *enoxaparin 0.75 mg/kg once daily in CrCl 10-30 mL/min*  *Prophylactic anticoagulation:*  *- UFH 5000 IU TID (if weight < 120 kg), or 7500 IU TID (if weight > 120 kg)*  *- enoxaparin 40 mg once daily (if weight < 120 kg), or 40 mg twice daily (if weight > 120 kg)* |
| *4* | *Bielza (2021)* | X | - | - | *Treatment: enoxaparin without information about dose or type*  *Control arm: no treatment* |
| *5* | *Billett (2020)* | X | X | - | *Therapeutic anticoagulation: enoxaparin or UFH without information about dose*  *Prophylactic anticoagulation: enoxaparin or UFH without information about dose* |
| *6* | *Bolzetta (2020)* | X | - | - | *Therapeutic anticoagulation: calciparin, fondaparinux, enoxaparine therapeutic dose (unspecified)*  *Prophylactic anticoagulation: calciparin, fondaparinux, enoxaparine prophylactic dose (unspecified)* |
| *7* | *Canoglu (2020)* | X | - | X | *Therapeutic anticoagulation: enoxaparin 1 mg/kg twice daily*  *Prophylactic anticoagulation: enoxaparin 0.5 mg/kg twice daily* |
| *8* | *Desai (2020)^4^* | X | - | - | *LMWH without information about dose*  *Control arm: no treatment* |
| *9* | *Di Castelnuovo (2021)* | X | - | - | *Therapeutic anticoagulation: LMWH therapeutic dose (unspecified)*  *Prophylactic anticoagulation: LMWH prophylactic dose (unspecified)* |
| *10* | *Falcone (2020)^8^* | X | - | - | *Treatment arm: LMWH prophylactic (40-40 mg daily) or full dose (40-60 twice daily)*  *Control arm: no treatment* |
| *11* | *Felldin (2020)^1^* | X | - | - | *Treatment arm: LMWH without information about dose*  *Control arm: no treatment* |
| *12* | *Ferguson (2020)* | X | X | - | *Treatment arm: therapeutic anticoagulation with continuous infusion of heparin dose-adjusted based on unfractionated heparin level or by subcutaneous 1 mg/kg twice daily or 1.5 mg/kg daily LMWH*  *Control arm: prophylaxis with enoxaparin 40 mg subcutaneously daily, enoxaparin 30 mg twice daily, enoxaparin 0.5 mg/kg twice daily, or heparin 5000 UI subcutaneously twice or 3 times daily* |
| *13* | *Hsu (2020)* | X | X | - | *Therapeutic anticoagulation: intravenous heparin, LMWH 1 mg/kg twice daily*  *Standard prophylactic anticoagulation:LMWH 40 mg once daily or UFH subcutaneous 5000 IU three times daily*  *High intensity prophylactic anticoagulation:LMWH 40 mg twice daily or UFH subcutaneous 7500 IU three times daily* |
| *14* | *Ionescu (2020)^5^* | X | X | - | *Full dose: >3-day*  *- UFH with at least one documented activated partial thromboplastin time in the anticoagulation range (≥45 seconds);*  *- enoxaparin at doses of 1 mg/kg twice daily or 1.5 mg/kg*  *once daily (while allowing for dose adjustment based on creatinine*  *clearance);*  *- intravenous argatroban infusion;*  *- fondaparinux at doses of 5-10 mg once daily (weight-based dosing);*  *- oral anticoagulants (warfarin, apixaban, rivaroxaban, dabigatran) prescribed prior to and continued throughout hospitalization.*  *Prophylactic anticoagulation:*  *-UFH at doses of 5000 units twice or three times daily;*  *- enoxaparin injection at doses of 30-40 mg once daily;*  *- fondaparinux at a dose of 2.5 mg once daily.* |
| *15* | *Jonmarker (2020)* | X | X | - | *Low-dose prophylactic anticoagulation: tinzaparin 2500-4500 IU, dalteparin 2500-5000 IU*  *Medium-dose prophylactic anticoagulation: tinzaparin > 4500 IU but < 175 IU/kg of body weight, dalteparin > 5000 IU but < 200 IU/kg of body weight*  *High-dose prophylactic anticoagulation: tinzaparin ≥ 175 IU/kg of body weight, dalteparin ≥ 200 IU/kg of body weight* |
| *16* | *Li (2020)* | X | X | X | *Treatment arm: UFH infusion from 8.4 units/kg/h up to 15.1 units/kg/h*  *Control arm: no treatment, or standard prophylaxis with subcutaneous UFH 5000 IU twice or three times daily, or enoxaparin 40 mg once daily* |
| *17* | *Llitjos (2020)* | X | - | - | *Therapeutic anticoagulation: LMWH or UFH therapeutic dose (unspecified)*  *Prophylactic anticoagulation: LMWH or UFH prophylactic dose (unspecified)* |
| *18* | *Lopes (2020)* | X | - | X | *Therapeutic anticoagulation:* *subcutaneous enoxaparin 1 mg/kg twice daily or intravenous UFH (to achieve a 0·3–0·7 IU/mL anti-Xa concentration)*  *Prophylactic anticoagulation: prophylactic dose enoxaparin or UFH (unspecified)* |
| *19* | *Martìnez-Botìa (2021)* | X | X | X | *Therapeutic anticoagulation: enoxaparin, bemiparin, fondaparinux (LMWH) therapeutic dose (unspecified)*  *Intermediate anticoagulation: LMWH intermediate dose (unspecified)*  *Prophylactic anticoagulation: LMWH prophylactic dose (unspecified)* |
| *20* | *Monforte (2020)^3^* | X | - | - | *Prophylactic: not defined*  *Control arm: no treatment* |
| *21* | *Motta (2020)* | X | - | - | *Prophylactic: enoxaparin 30-40 mg subcutaneously daily or intravenous heparin titrated on aPTT time*  *Therapeutic anticoagulation: enoxaparin 1 mg/kg subcutaneously twice daily or 1.5 mg/kg subcutaneously daily or based on renal function, or intravenous heparin titrated on aPTT time* |
| *22* | *Nadeem (2020)* | X | X | X | *Treatment: fixed-dose, increasing dose, decreasing dose, variable quantity of enoxaparin* |
| *23* | *Nadkarni (2020)^31^* | X | X | - | *Prophylactic: subcutaneous unfractionated heparin, LMWH once daily, or apixaban (2.5 mg twice a day or 5 mg daily in patients ≤75 years) were administered*  *Full dose: continuous intravenous infusions of bivalirudin, argatroban or unfractionated heparin (UFH), high dose LMWH (specifically enoxaparin 1 mg/kg twice daily or 1.5 mg/kg daily), apixaban 5mg twice daily, rivaroxaban or dabigatran* |
| *24* | *Pesavento (2020)* | - | X | - | *Prophylactic anticoagulation: UFH 5000 IU three times daily, or LMWH 40 mg daily, or fondaparinux 2.5 mg daily*  *Treatment anticoagulation: LMWH 0.5 mg/kg to 1 mg/kg twice daily, or fondaparinux 7.5 mg daily* |
| *25* | *Qin (2021)* | X | - | - | *Treatment arm: prophylactic dose (3000-5000 IU daily) or therapeutic dose (100 IU/kg twice daily) subcutaneous LMWH* |
| *26* | *Rentsch (2021)* | X | X | - | *Treatment arm:*  *- prophylactic dosage of heparin (5000 IU subcutaneously twice daily or three times daily), or enoxaparin (40 mg once daily or 30 mg twice daily), or fondaparinux (2.5 mg daily) or dalteparin (2500-5000 IU daily)*  *- therapeutic dosage of heparin (titrated on PTT), enoxaparin (>40 mg daily), fondaparinux (5 to 10 mg daily), dalteparin (>5500 IU twice daily)*  *Control arm: no treatment* |
| *27* | *Rodriguez-Nava (2021)* | X | - | - | *Treatment arm:*  *- prophylactic enoxaparin or heparin (without information of dosage)*  *- therapeutic enoxaparin or heparin (without information on dosage)*  *Control arm: no treatment* |
| *28* | *Shen (2020)* | X | - | - | *Treatment arm: enoxaparin 40 mg daily or twice daily*  *Control arm: no treatment* |
| *29* | *Shi*  *(2020)*^10^ | - | - | X | *Treatment arm: Prophylactic dose of enoxaparin/nadroparin/LMWH*  *Control arm: no treatment* |
| *30* | *Tang (2020)^7^* | X | - | - | *Treatment arm: UFH or LMWH for 7 days or longer without information about dose or type*  *Control arm: no treatment* |
| *31* | *Ugur (2021)* | X | - | - | *Treatment arm: prophylactic dose LMWH (unspecified)*  *Control arm: no treatment* |
| *32* | *Yormaz (2020)^11^* | X | - | X | *Prophylactic arm: LMWH dose of 4000 IU/day, for 7 days*  *No anticoagulant drugs other than heparin were utilized for seven days or longer in the research patients*  *Control arm: no treatment* |
| *33* | *Yu (2021)* | X | X | - | *Prophylactic arm: prophylactic dose of anticoagulation, without information about dose or type*  *Therapeutic arm: therapeutic dose of anticoagulation, without information about dose or type* |

LMWH: low molecular weight heparin, TPA: tissue plasminogen activator, UFH: unfractionated heparin

**Supplementary Figure 1. Funnel plots for each endpoint according to overall analysis (Column A), prophylactic dose analysis (Column B), full dose analysis (Column C) against no treatment, and comparison between prophylactic and full dose (Column D)**

**
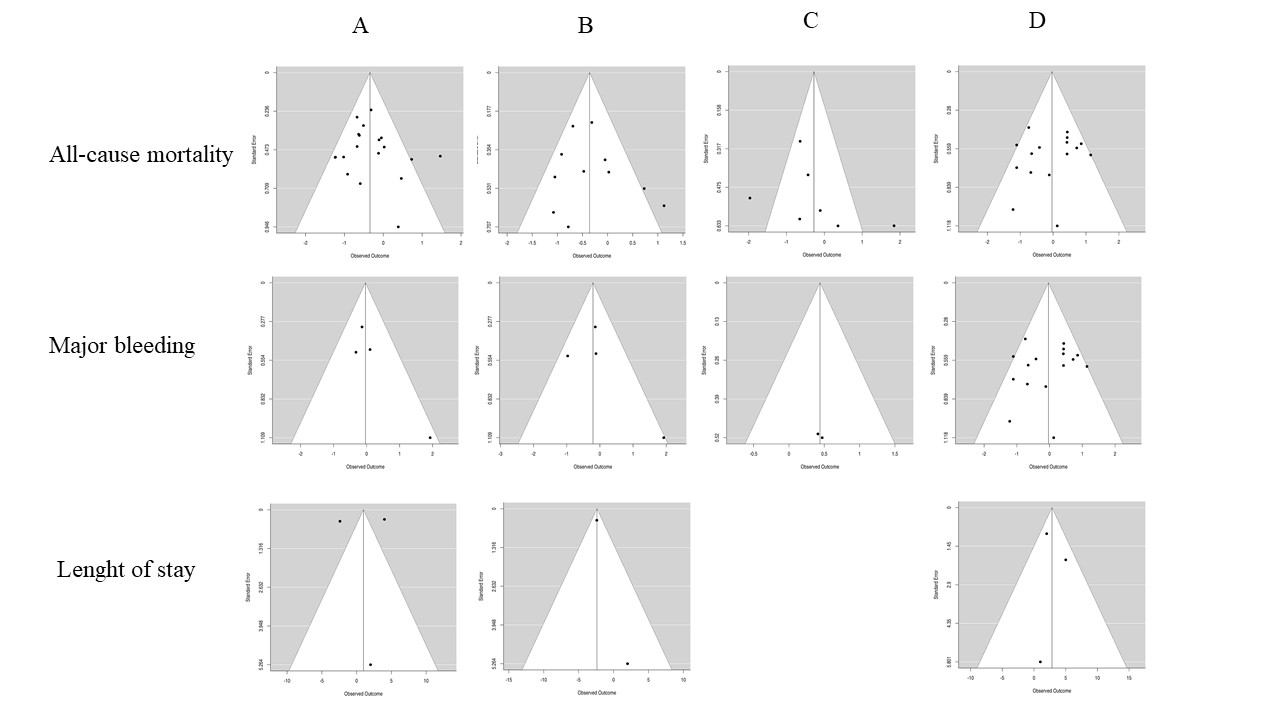
**

**Supplementary Figure 2. Major bleeding risk in patients treated with heparin: overall analysis (Panel A), sensitivity analysis of prophylactic dose (Panel B) and full dose (Panel C) against no treatment. Full dose heparin versus prophylactic heparin was reported in Panel D**

**A**


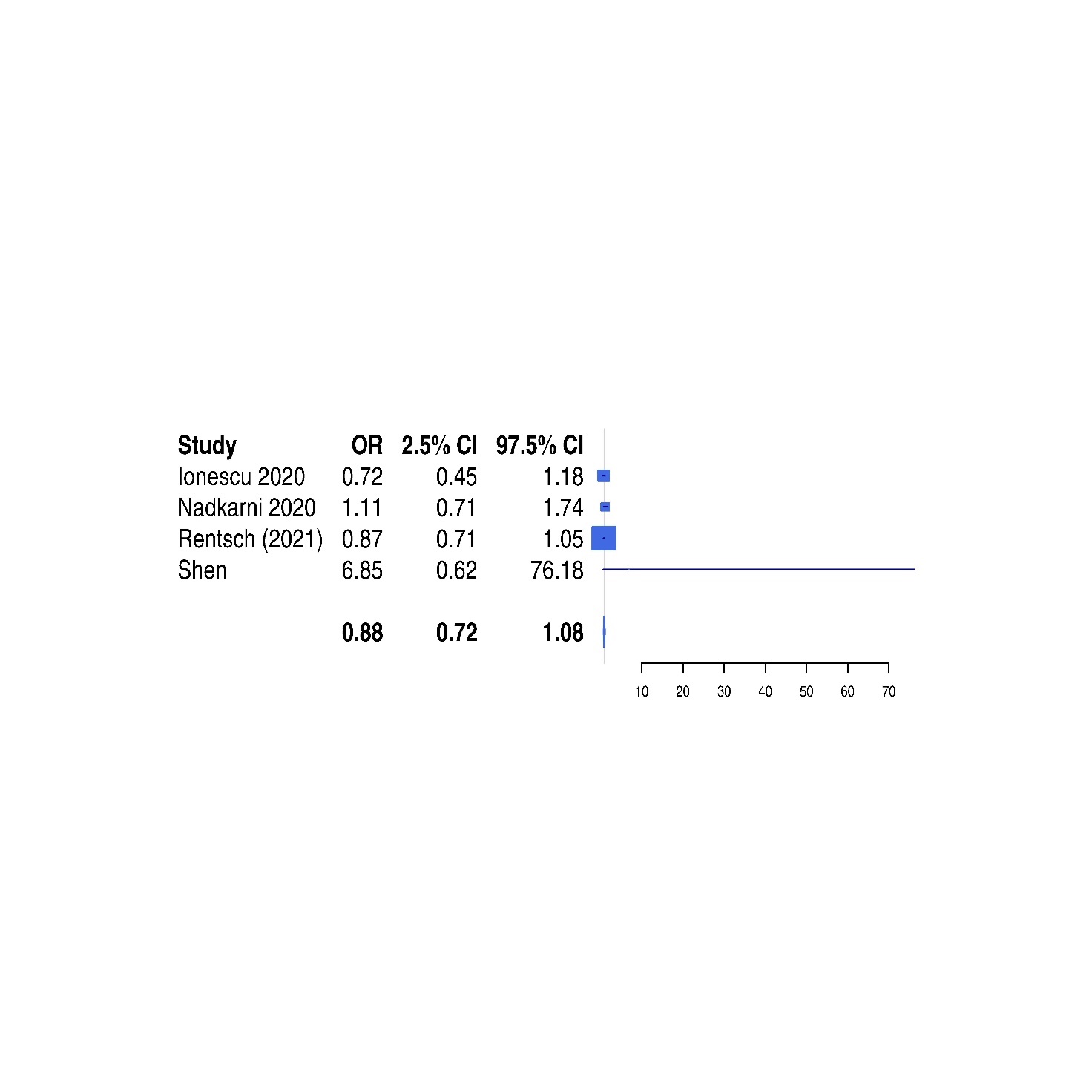


**B**


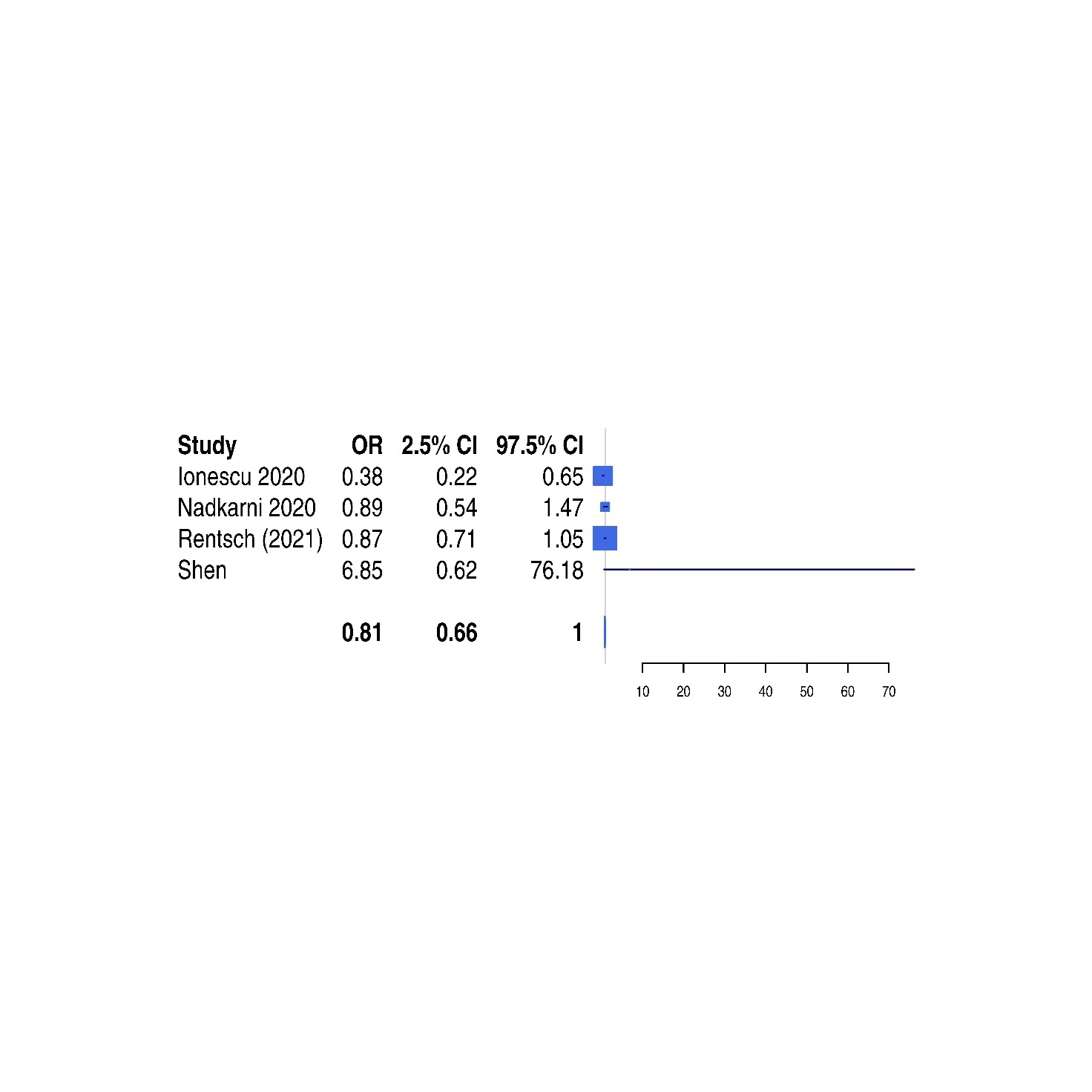


**C**


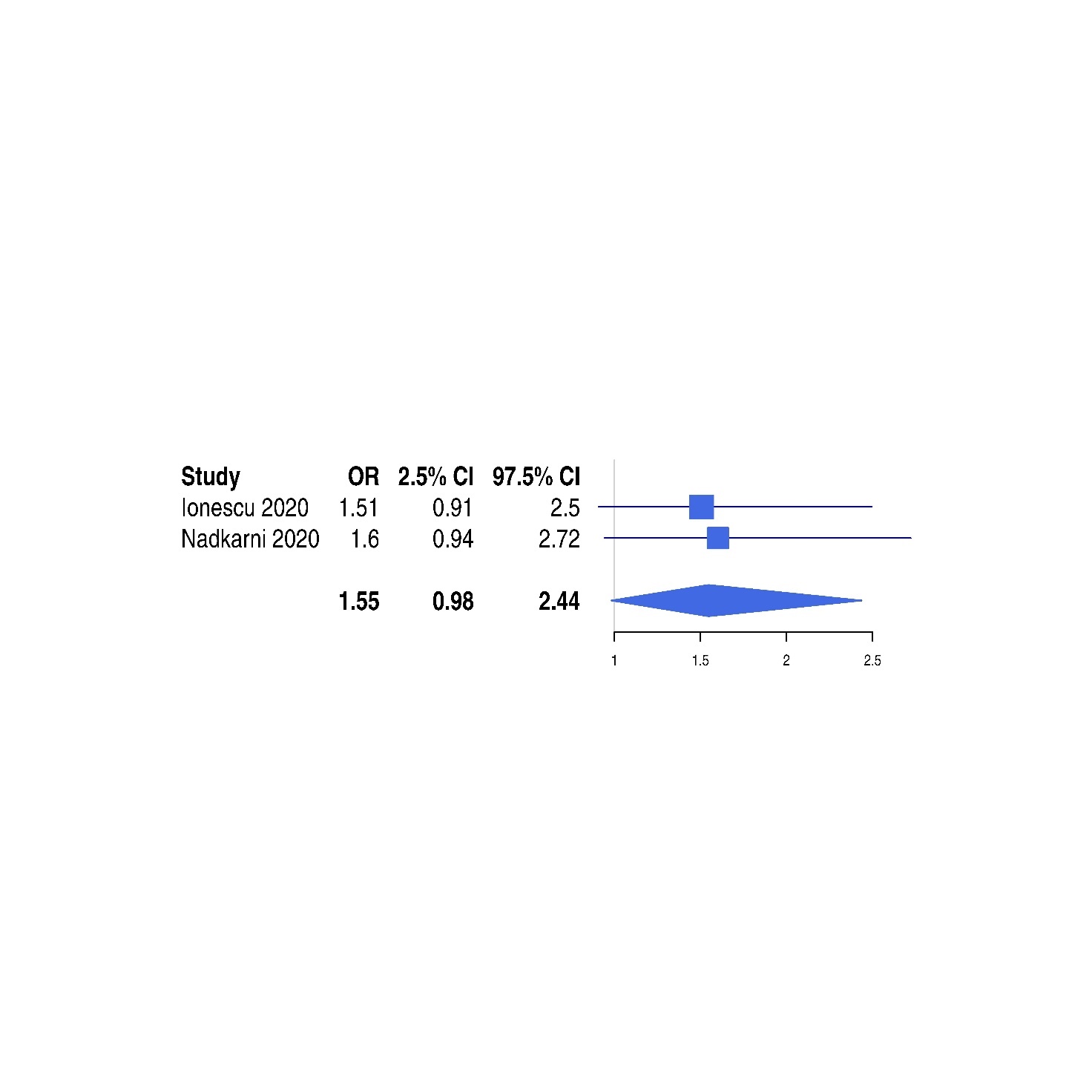


**D**


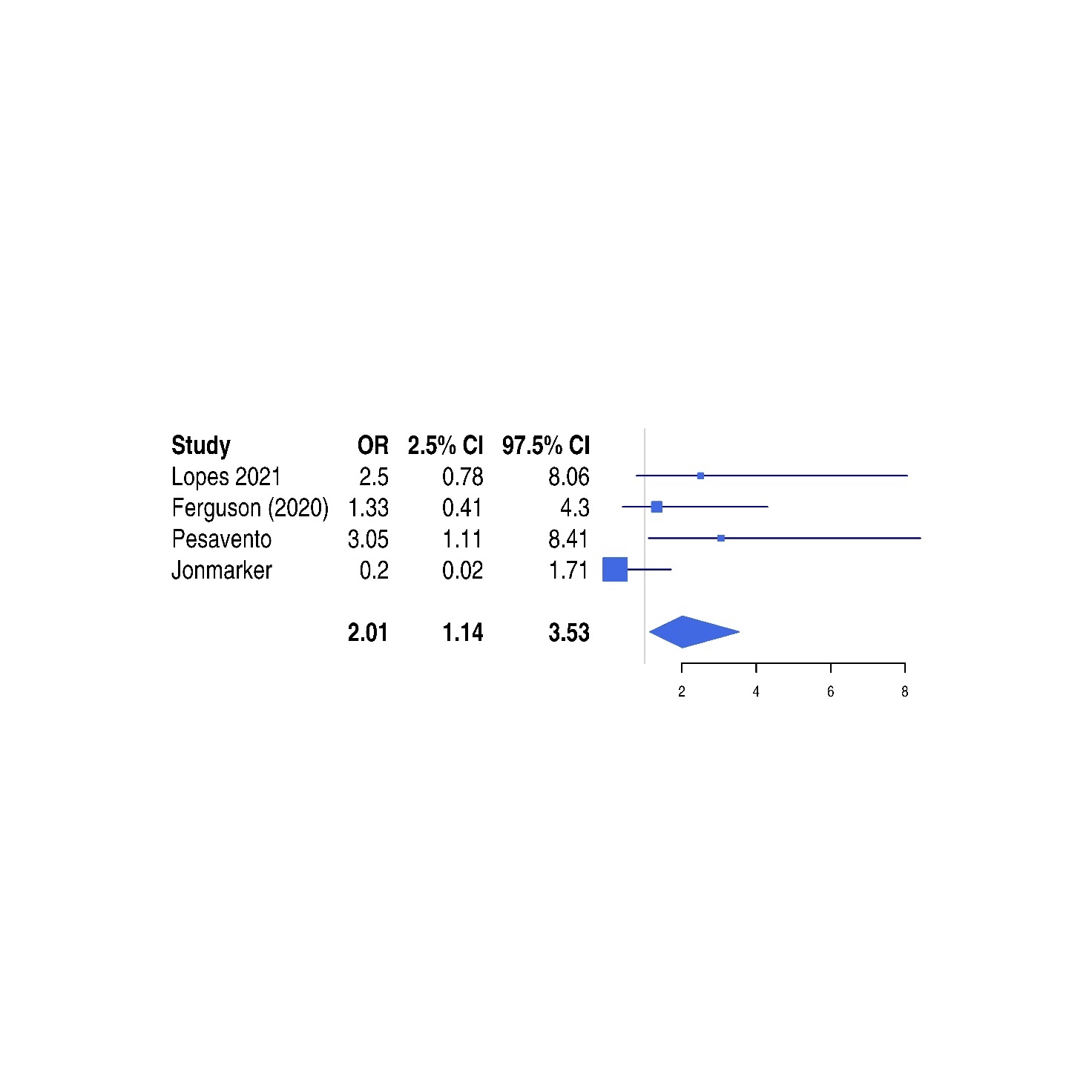


**Supplementary Figure 3. Length of hospital stay of patients treated with heparin: overall analysis (Panel A), subgroup of prophylactic dose (Panel B) against no treatment. Full dose heparin versus prophylactic heparin was reported in Panel C**

**A**


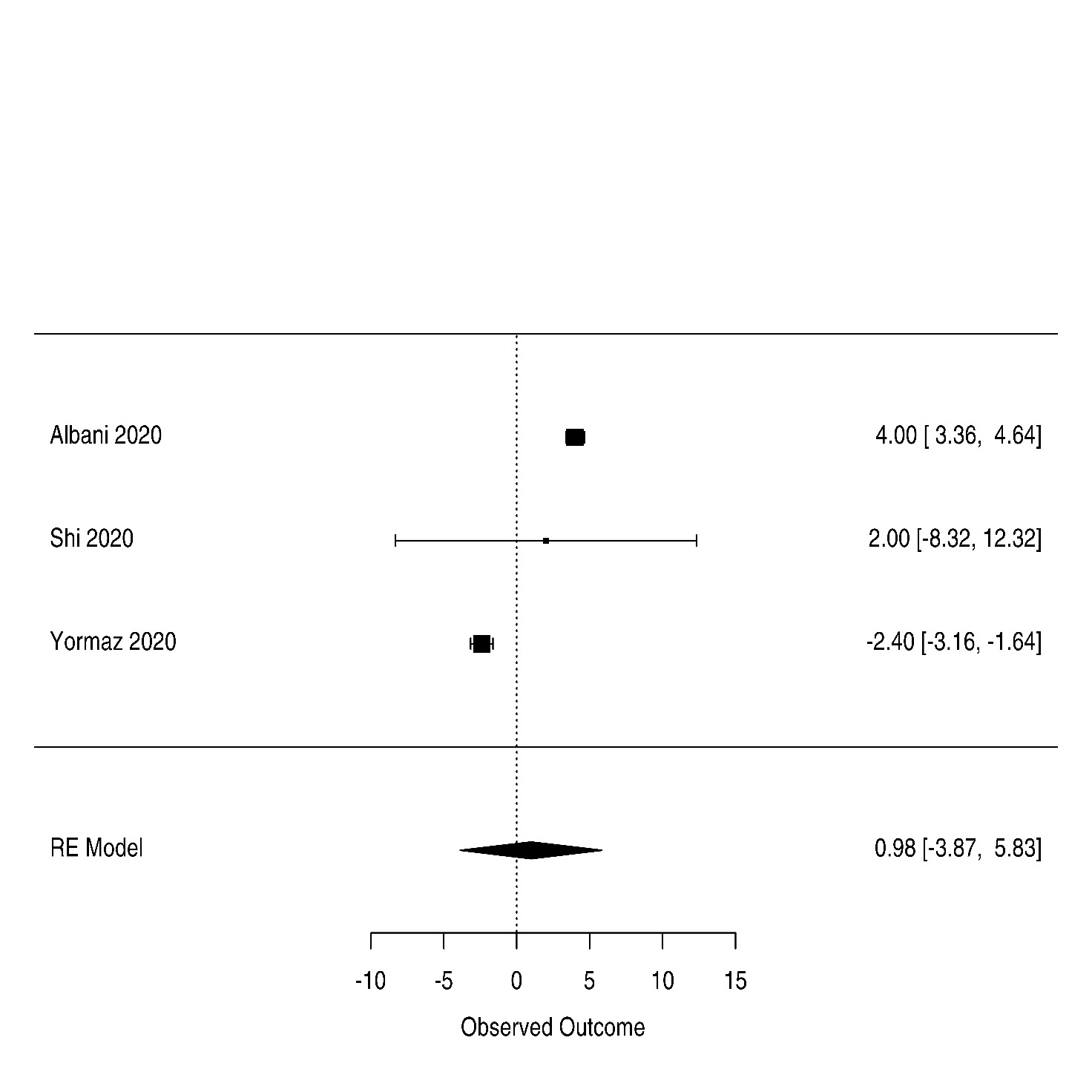


**B**


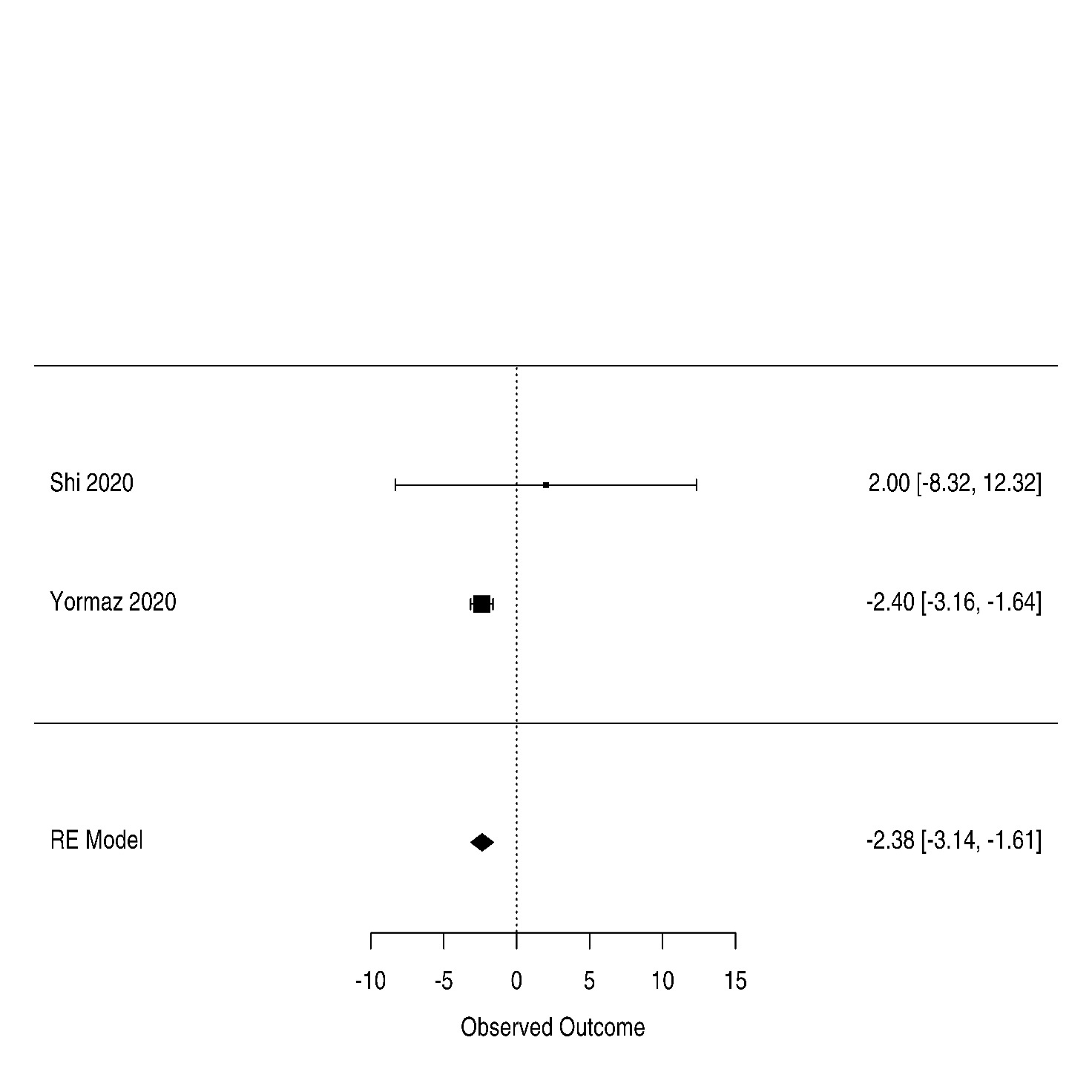


**C**


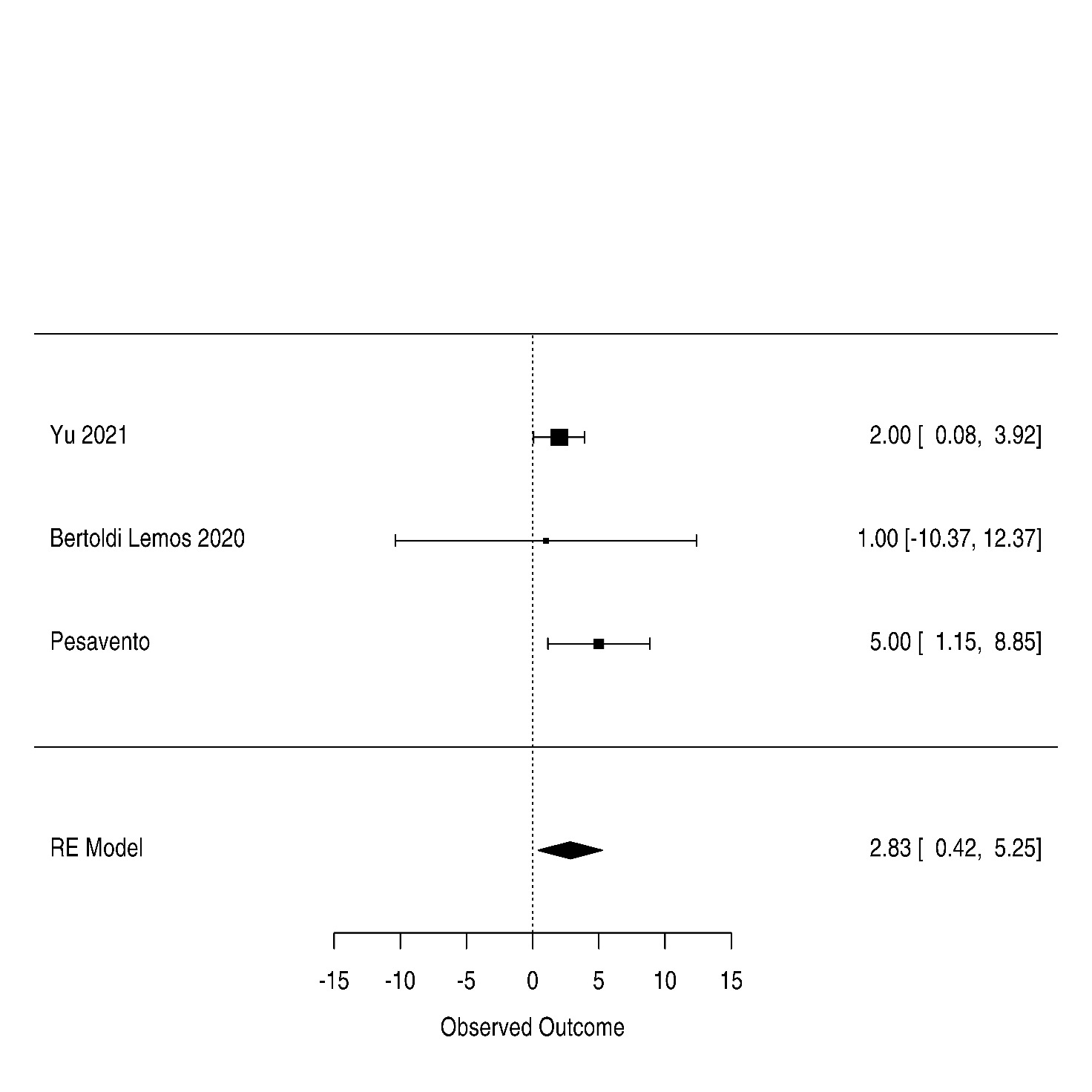


**Reference**

1. Felldin M, Softeland JM, Magnusson J, et al. Initial Report From a Swedish High-Volume Transplant Center After The First Wave of The COVID-19 Pandemic. *Transplantation.* 2020.

2. Albani F, Sepe L, Fusina F, et al. Thromboprophylaxis with enoxaparin is associated with a lower death rate in patients hospitalized with SARS-CoV-2 infection. A cohort study. *EClinicalMedicine.* 2020;27:100562.

3. d'Arminio Monforte A, Tavelli A, Bai F, et al. The importance of patients' case-mix for the correct interpretation of the hospital fatality rate in COVID-19 disease. *Int J Infect Dis.* 2020;100:67-74.

4. Desai A, Voza G, Paiardi S, et al. The role of anti-hypertensive treatment, comorbidities and early introduction of LMWH in the setting of COVID-19: A retrospective, observational study in Northern Italy. *Int J Cardiol.* 2020.

5. Ionescu F, Jaiyesimi I, Petrescu I, et al. Association of anticoagulation dose and survival in hospitalized COVID-19 patients: A retrospective propensity score-weighted analysis. *Eur J Haematol.* 2020.

6. Ayerbe L, Risco C, Ayis S. The association between treatment with heparin and survival in patients with Covid-19. *J Thromb Thrombolysis.* 2020;50(2):298-301.

7. Tang N, Bai H, Chen X, Gong J, Li D, Sun Z. Anticoagulant treatment is associated with decreased mortality in severe coronavirus disease 2019 patients with coagulopathy. *J Thromb Haemost.* 2020;18(5):1094-1099.

8. Falcone M, Tiseo G, Barbieri G, et al. Role of Low-Molecular-Weight Heparin in Hospitalized Patients With Severe Acute Respiratory Syndrome Coronavirus 2 Pneumonia: A Prospective Observational Study. *Open Forum Infect Dis.* 2020;7(12):ofaa563.

9. Billett HH, Reyes-Gil M, Szymanski J, et al. Anticoagulation in COVID-19: Effect of Enoxaparin, Heparin, and Apixaban on Mortality. *Thromb Haemost.* 2020;120(12):1691-1699.

10. Shi C, Wang C, Wang H, et al. The Potential of Low Molecular Weight Heparin to Mitigate Cytokine Storm in Severe COVID-19 Patients: A Retrospective Cohort Study. *Clin Transl Sci.* 2020;13(6):1087-1095.

11. Yormaz B, ErgUn D, B TU, ErgUn R, Arslan U, Kanat F. The impact of the "low molecular weight heparin" administration on the clinical course of COVID-19 disease. *Turk J Med Sci.* 2020.

12. Yu B, Gutierrez VP, Carlos A, et al. Empiric use of anticoagulation in hospitalized patients with COVID-19: a propensity score-matched study of risks and benefits. *Biomark Res.* 2021;9(1):29.

13. Pesavento R, Ceccato D, Pasquetto G, et al. The hazard of (sub)therapeutic doses of anticoagulants in non-critically ill patients with Covid-19: The Padua province experience. *J Thromb Haemost.* 2020;18(10):2629-2635.

14. Martinez-Botia P, Bernardo A, Acebes-Huerta A, et al. Clinical Management of Hypertension, Inflammation and Thrombosis in Hospitalized COVID-19 Patients: Impact on Survival and Concerns. *J Clin Med.* 2021;10(5).

15. Rentsch CT, Beckman JA, Tomlinson L, et al. Early initiation of prophylactic anticoagulation for prevention of coronavirus disease 2019 mortality in patients admitted to hospital in the United States: cohort study. *BMJ.* 2021;372:n311.

16. Rodriguez-Nava G, Yanez-Bello MA, Trelles-Garcia DP, et al. Clinical Characteristics and Risk Factors for Death of Hospitalized Patients With COVID-19 in a Community Hospital: A Retrospective Cohort Study. *Mayo Clin Proc Innov Qual Outcomes.* 2021;5(1):1-10.

17. Bielza R, Sanz J, Zambrana F, et al. Clinical Characteristics, Frailty, and Mortality of Residents With COVID-19 in Nursing Homes of a Region of Madrid. *J Am Med Dir Assoc.* 2021;22(2):245-252 e242.

18. Ugur M, Adiyeke E, Recep E, Bakan N, Yiyit N. Aggressive Thromboprophylaxis Improves Clinical Process and Decreases the Need of Intensive Care Unit in Covid-19. *Pak J Med Sci.* 2021;37(3):668-674.

19. Di Castelnuovo A, Costanzo S, Antinori A, et al. Heparin in COVID-19 Patients Is Associated with Reduced In-Hospital Mortality: the Multicenter Italian CORIST Study. *Thromb Haemost.* 2021.

20. Hsu A, Liu Y, Zayac AS, Olszewski AJ, Reagan JL. Intensity of anticoagulation and survival in patients hospitalized with COVID-19 pneumonia. *Thromb Res.* 2020;196:375-378.

21. Qin W, Dong F, Zhang Z, et al. Low molecular weight heparin and 28-day mortality among patients with coronavirus disease 2019: A cohort study in the early epidemic era. *Thromb Res.* 2021;198:19-22.

22. Shen L, Qiu L, Liu D, et al. The Association of Low Molecular Weight Heparin Use and In-hospital Mortality Among Patients Hospitalized with COVID-19. *Cardiovasc Drugs Ther.* 2021.

23. Llitjos JF, Leclerc M, Chochois C, et al. High incidence of venous thromboembolic events in anticoagulated severe COVID-19 patients. *J Thromb Haemost.* 2020;18(7):1743-1746.

24. Li M GS, Nyabera A. Kondaveeti R., Hammudeh Y., Gonzalez C., Trandafirescu T., Penumadu A., Lopez R., Sahibzada A., De La Cruz A., Rahman H. Continuous Infusion Low-Dose Unfractionated Heparin for the Management of Hypercoagulability Associated With COVID-19. *Journal of Pharmacy Practice.* 2020.

25. Motta JK, Ogunnaike RO, Shah R, et al. Clinical Outcomes With the Use of Prophylactic Versus Therapeutic Anticoagulation in Coronavirus Disease 2019. *Crit Care Explor.* 2020;2(12):e0309.

26. Bolzetta F, Maselli M, Formilan M, et al. Prophylactic or therapeutic doses of heparins for COVID-19 infection? A retrospective study. *Aging Clin Exp Res.* 2021;33(1):213-217.

27. Nadeem R, Thomas SJ, Fathima Z, et al. Pattern of anticoagulation prescription for patients with Covid-19 acute respiratory distress syndrome admitted to ICU. Does it impact outcome? *Heart Lung.* 2021;50(1):1-5.

28. Canoglu K, Saylan B. Therapeutic dosing of low-molecular-weight heparin may decrease mortality in patients with severe COVID-19 infection. *Ann Saudi Med.* 2020;40(6):462-468.

29. Ferguson J, Volk S, Vondracek T, Flanigan J, Chernaik A. Empiric Therapeutic Anticoagulation and Mortality in Critically Ill Patients With Respiratory Failure From SARS-CoV-2: A Retrospective Cohort Study. *J Clin Pharmacol.* 2020;60(11):1411-1415.

30. Jonmarker S, Hollenberg J, Dahlberg M, et al. Dosing of thromboprophylaxis and mortality in critically ill COVID-19 patients. *Crit Care.* 2020;24(1):653.

31. Nadkarni GN, Lala A, Bagiella E, et al. Anticoagulation, Bleeding, Mortality, and Pathology in Hospitalized Patients With COVID-19. *J Am Coll Cardiol.* 2020;76(16):1815-1826.
